# Automated Diagnosis of Coronary Artery Disease: A Review and Workflow

**DOI:** 10.1155/2018/2016282

**Published:** 2018-02-04

**Authors:** Qurat-ul-ain Mastoi, Teh Ying Wah, Ram Gopal Raj, Uzair Iqbal

**Affiliations:** Faculty of Computer Science and Information Technology, University of Malaya, Kuala Lumpur 50603, Malaysia

## Abstract

Coronary artery disease (CAD) is the most dangerous heart disease which may lead to sudden cardiac death. However, CAD diagnoses are quite expensive and time-consuming procedures which a patient need to go through. The aim of our paper is to present a unique review of state-of-the-art methods up to 2017 for automatic CAD classification. The protocol of review methods is identifying best methods and classifier for CAD identification. The study proposes two workflows based on two parameter sets for instances A and B. It is necessary to follow the proper procedure, for future evaluation process of automatic diagnosis of CAD. The initial two stages of the parameter set A workflow are preprocessing and feature extraction. Subsequently, stages (feature selection and classification) are same for both workflows. In literature, the SVM classifier represents a promising approach for CAD classification. Moreover, the limitation leads to extract proper features from noninvasive signals.

## 1. Introduction

Cardiovascular diseases (CVDs) are the major reasons for mortality around the world. According to World Health Organization (WHO), approximately 17.7 million people died in 2015, representing to 31% of all global death [1]. However, European Heart Network and European Society of Cardiology estimate that over 4 million people died from CVDs in Europe and 1.9 million people died in European Union (EU) which are 47% and 40% deaths, respectively [2]. We all know that the human heart is the most crucial and hardest working organ of the body that combines with blood vessels to form the whole cardiovascular system [3]. CVD is caused by disorders of the heart and blood vessels which result in coronary artery disease (CAD), heart failure, cardiac arrest, and sudden cardiac death [4].

In order to diagnose positive symptoms of CAD, medical specialists prescribed various tests such as angiography, nuclear scan, and C-reactive protein test which are quite expensive and require technical experts [5]; therefore, researchers are seeking interest to develop a less expensive and an effective alternative to the costly prescribed test. In literature, automatic CAD-diagnosing techniques using machine learning algorithms and data mining method have been developed [5–8] for reducing the medical specialist's efforts and time and save patients' lives and cost. Furthermore, this paper describes a unique review of existing studies found in the literature regarding the identification of CAD symptoms from signal recording and CAD classification using other clinical parameters.

**Figure 1:**
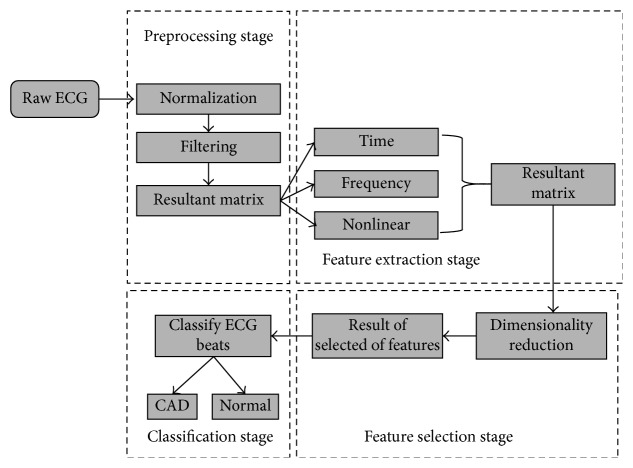
Workflow for parameter set A.

**Figure 2:**
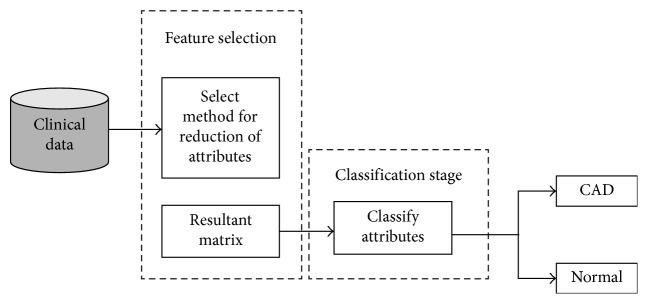
Workflow for parameter set B.

The procedure of identifying and classifying CAD diagnosis automatically from noninvasive data is required to follow a proper work direction. In literature, there are two types of studies found, some studies used signal recording to identify CAD symptoms, for instance, electrocardiograph (ECG), photoplethysmography (PPG), and phonocardiography (PCG), and other studies used clinical parameters like age, blood pressure, and smoking habit to classify CAD patients. Therefore, our study proposed two workflows which help to guide the evaluation process of future works (see Figures 1 and 2).

The initial two steps of Figure 1 are preprocessing and feature extraction. These stages are widely used in the literature [7–10] which used signal recording (noninvasive data) to identify CAD symptoms. The techniques utilized in first two stages directly influence the classification results; therefore, it is necessary to choose the technique carefully. Subsequently, the remaining two stages of workflows (feature reduction and classification) are the same for both studies in Figures 1 and 2. Note that workflows for evaluation process of future works are a significant contribution to this review work. In the recent studies, we find a review of signal recording pattern recognition and classification [11] techniques based on nonlinear transformation. Rajkumar et al. [12] performed an extensive review and comparative analysis of methods used for CAD classification. In specific, they [12] did not focus on the workflows for the evaluation process of future direction and their study only reviews the state-of-the-art classifiers. However, our study focuses on more up-to-date literature review. Furthermore, our study performs a special review of existing classification methods.

The rest of the paper is organized as follows: Section 2 gives an explanation regarding databases for CAD diagnosis. The review of the methodology procedures is discussed in Section 3. Finally, discussion and conclusion are summarized in Section 4 and Section 5, respectively.

## 2. Data Acquisition

Various databases are developed for the heart disease and arrhythmia classification which allow the researcher to evaluate their methods on the standardized database. There are few datasets which are more commonly used in studies for CAD and its risk identification.

### 2.1. The Long-Term ST Database

The benchmark database of PhysioNet contains 86 lengthy ECG recordings of 80 human subjects. In literature, studies consider 23 subjects of this database which are only affected by CAD [13].

### 2.2. UCI Repository of Machine Learning Database

The database was collected from the Cleveland Clinic Foundation. It has 76 parameters, out of which only 14 parameters were selected for use. The selected attributes represent the clinical and noninvasive test results of 303 patients who are undergoing angiography. Removing the cases containing missing values, 270 cases were considered in studies, out of which 120 cases were identified as patients with CHD while 150 cases were diagnosed as patients without CHD [14].

### 2.3. IQRAA Hospital, Calicut, Kerala, India

Some of the studies use ECG signals of 10 CAD patients from the IQRAA Hospital, Calicut, Kerala, India [7]. BIOPAC TM equipment was used to record the ECG signals at a sampling rate of 500 Hz [15]. All the CAD subjects participated in the studies were on similar medication. The age of all the subjects was under the ranges between 40 and 70 years.

### 2.4. Multiparameter Intelligent Monitoring in Intensive Care (MIMIC II)

The MIMIC II database [16] contains two types of ICU patient records: waveform dataset and clinical dataset. The waveform dataset contains physiological signal recording (such as ECG, PPG, and arterial blood pressure (ABP)), and clinical dataset contains clinical data which are collected by hospital staff.

## 3. Methodology Procedure

CAD is caused by atherosclerosis of the coronary arteries that leads to formation of barrier on the blood flow to the heart which may be diagnosed using clinical data of the patient such as blood pressure, age, gender, smoking habit, and random blood sugar and identifies symptoms of CAD using ECG. However, existing studies follow different procedures for diagnosing CAD using machine learning methods and data mining techniques. However, we proposed a common workflow for the new researcher for their work evaluation which is described in Figures 1 and 2. Moreover, Figures 1 and 2 are based on those studies which utilize raw signals as a dataset and clinical dataset for diagnosing CAD, respectively. Figure 1 describes that CAD classification using raw signal dataset has more steps to diagnose as compared to Figure 2. Furthermore, our study reviewed each stage separately.

### 3.1. Preprocessing

Contaminated recordings were the major problem of detecting coronary artery disease; however, studies used a different method to preprocess data prior to feature extraction step. This section reviews those preprocessing techniques which were used in the context of coronary artery disease detection.

Davari Dolatabadi et al. [8] and Patidar et al. [9] used a low-pass filter and a high-pass filter with a cutoff frequency for removing 20 Hz noise and 0.3 Hz noise, respectively, whereas a 50 Hz notch filter is used to remove power source interference, and this filter is also called band-rejection filter. However, the Pan–Tompkins [17] algorithm is used to analyze the R peak for the measurement of two consecutive beats RR interval and QRS detection. The Pan–Tompkins method is widely used in literature because it is simple and easy to implement.

Kumar et al. [7] used baseline wander for low and high cutoff frequency of 0.3 Hz and 15 Hz, respectively. Similarly, the study used notch filter and Pan–Tompkins methods to eliminate 50 Hz cutoff frequency and identify R-peaks separately.

Ukil et al. [18] proposed the methodology for cleaning PPG signals for CAD detection. The multistage method is used to analyze the presence of noise in the signal. In the first part, the study used dynamic time wrapping technique for segmentation. Secondly, the Hampel filter is used to remove the noise from the signal.

Contrasting with previous mentioned techniques, discretization techniques were presented in [19] for the parameter intervals. Discretization is a process of dividing the continuous parameter in a discretized variable for classification of coronary artery disease parameters. However, this technique had a direct impact on the performance of classification method which is widely used for data analysis in [20, 21].

### 3.2. Feature Extraction

This stage is one of the major keys to the success route of CAD detection. The feature extraction is the process of revealing clinical features from the signal's morphology in the time and frequency domains. This phase of classification is only used by those studies which refer raw ECG signal as a dataset.

According to [8], time, frequency, and nonlinear dynamic features are used to demonstrate CAD patient and non-CAD patients. For the measurement of frequency domain features, the study employed autoregressive (AR) modeling-based method to calculate power spectrum density; AR spectrum is the most popular method for HRV analysis, and this algorithm has the capability to be factorized into separate spectral components [22]. AR model is more complex, and it has the contingency of negative components in spectral factorization. Subsequently, for time domain calculation, the authors used statistical features and geometrical features like SD, RMSSD, and HRV triangular index, respectively.

Giri et al. [10] employed the wavelet transform for uncovering the time- and frequency-localizing features from the signals. The wavelet transform is the most common technique which is widely used by many studies in literature, the main advantage of this technique is that it extracts time-frequency scale features. The study used a resultant matrix of wavelet coefficient feed classifier for CAD detection.

Patidar et al. [9] proposed the tunable-Q wavelet transform (TQWT) [23] for correntropy features for CAD; TQWT is a process of analyzing the oscillatory signal using three parameters: *Q*, total over-sampling rate or redundancy denoted as *r*, and the number of levels of decomposition denoted as *J*. The study describes that the wavelet transform technique is simple to extract time-frequency scale features from the HRV signals, whereas correntropy-based nonlinear features can be used to measure the CAD changes in the heart rate signals.

The flexible analytic wavelet transform [24] technique is used to decompose HRV signals to analyze adjustable parameters *a*, *b*, *c*, *d*, and *β*. However, *a* and *b* are the sampling parameters of up and down for the low-pass channel, respectively, whereas *c* and *d* are the sampling parameters of up and down for the high-pass channel, respectively [7].

### 3.3. Feature Selection

Feature selection is the process of choosing only selected parameters from the set of features for improving the classification accuracy. This phase of classification is used by both the workflows which are described in Figures 1 and 2. We analyzed that feature selection is a prerequisite requirement of studies; either it used raw ECG signal or other clinical data.

According to [8, 9], principal component analysis (PCA) is the most valuable technique for analyses of relevant features. PCA is a statistical technique which is used as a dimensionality reduction technique for all types of analyses, and this method varies from neuroscience to computer graphics because it is simple and efficient. The study used PCA for its feature selection technique to analyze nonlinear features and time-frequency scale features in the context of CAD detection.

Giri et al. [10] proposed three-dimensionality reduction techniques, namely, PCA, linear discriminant analysis (LDA), and independent component analyses (ICA). The study used PCA for the calculation of eigenvectors for projecting the actual data into the directions of sorted eigenvalues, whereas ICA is another feature reduction method which transforms the multivariate random signal into a signal having components that are mutually independent. However, LDA provides the highest separation between the classes present in the feature set.

Feature selection technique is used to detect relevant attributes which lead to classifier accuracy. Marateb et al. [19] used two supervised methodologies for feature selection prior to diagnose coronary artery disease, the multiple logistic regression (MLR) [25], which was used to select statistically significant features, and deterministic approach sequential forward search (SFS) [26], while using SFS the classifier starts with an empty set and added features until the accuracy improved.

According to [27, 28], PCA is used for identifying the patterns of data and highlighting their similarities and differences. In contrast, PCA algorithm never uses the information to which signal belongs to which class. Therefore, the PCA directions represent the entire data irrespective of their class labels. These directions are not guaranteed to provide maximum separation between the classes [10].

### 3.4. Learning Methods

Once the set of features has been extracted and selected from the dataset, techniques can be built using the machine learning techniques and data mining domains for coronary artery disease classification.

**Figure 3:**
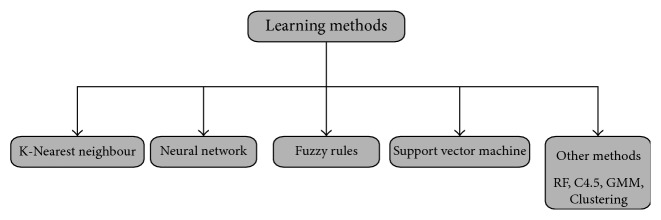
State-of-the-art classifiers.

The four most famous techniques which are depicted in Figure 3 are frequently found in literature to identify coronary artery disease detection which is k-nearest neighbor (KNN) [10, 29], neural network (NN) [30, 31], support vector machine (SVM) [7–9, 27], and fuzzy rule-based learning technique (FR) [19, 32]. It is noted that these methods are aiming the coronary artery disease classification. However, due to their importance for coronary artery disease classification, we discussed these four classifiers (KNN, NN, SVM, and FR) in the next sections (3.4.1, 3.4.2, 3.4.3, and 3.4.4), and other techniques are discussed in Section 3.4.5.

#### 3.4.1. K-Nearest Neighbor (KNN)

KNN is one of the most popular classifiers in machine learning field for coronary artery disease detection. KNN is also referred to as nonparametric because this technique does not use any assumptions on the data distribution, and hence it is called nonparametric. The study used KNN to compute [10] the technique for automatic classification of coronary artery disease. However, the author reveals that KNN provides better accuracy while performing linear discriminant analysis than SVM and NN.

Rajkumar and Reena [33] performed the experiment on the dataset to diagnose CAD using KNN and the study described that the KNN classifier obtained 45.67% accuracy only. Subsequently, Gilani et al. [34] performed F1 score comparison with several classifiers and they found that the KNN classifier performed best among seven classifiers in their study. Moreover, the authors also claim that KNN is not suitable for implementation in a low power or in a real-time application due to the high computational complexity during classification of a dataset.

The researchers in [35] have shown that KNN classifier obtained better accuracy measurement than SVM classifier for heart anomaly detection. Similarly, Faziludeen and Sankaran [36] used KNN and SVM for feature selection method with improved F-score measurement, while during this process, the result declared that KNN calculates best feature selection as compared to SVM.

#### 3.4.2. Neural Network (NN)

NN is a powerful classifier which is widely used in various fields of medical research because it is easy to implement and simple. NN is based on structure and functions of biological neural networks which bargain with neurons. This algorithm computes a solution in the similar way that the human brain works. In literature, we observed that NN is successfully implemented in the prediction of cardiac abnormalities [37].

Kim and Kang [30] proposed neural network classifier in the context of early diagnosing coronary artery disease risk prediction from the dataset. However, the study found that their proposed method with neural network is better than the Framingham risk score.

Kurt et al. [38] present a comparative study on classification techniques, namely, logistic regression, regression tree, and neural networks, for predicting coronary artery disease. NN is based on supervised learning methods, so it requires a desired response to be trained. Furthermore, the study found that NN outer performed among other classifiers.

Mitra and Samanta [39] suggested that NN usually solves multiple problems in the field and it is efficiently used in the large dataset as well but the limitation of the neural network is to retrain the method several times for accuracy. However, many studies used NN with other classifiers in order to obtain more accuracy in prediction. Güler and Übeyli [40] present NN with the generic method from a more sophisticated form of cross-validation. Furthermore, Osowski et al. suggested in [41] that the combination of classifier with the neural network not only reduces the overall error in the NN but also reduces the impact of false negative values in the testing phase.

#### 3.4.3. Support Vector Machine (SVM)

The SVM is based on statistical learning techniques which deal with the highly nonlinear network. SVM facilitates to classify unobservable patterns from the dataset [42]. According to [43], SVM is based on structural risk minimization; therefore, it renders more generalization than that of other traditional learning systems involving empirical risk minimization.

Davari Dolatabadi et al. [8] employed an SVM classifier to optimize the two parameters, namely, cost and sigma (*r*), for controlling the overfitting of the model and the degree of nonlinearity of the model, respectively. However, the study reveals that the proposed method uses less number of parameters to obtain the accuracy of 99.2%.

Giri et al. [10] performed an experiment on least square-SVM (LS-SVM) or it also may refer to an improved version of SVM or fastest technique than SVM. The study used LS-SVM to train and test the parameters of the CAD risk index. The study successfully classifies the normal and CAD patient from the dataset with the obtained accuracy of 99.72%.

Banerjee et al. [44] used SVM with Gaussian RBF kernel for classifying the CAD patients from the fingertip PPG signal. Their main objective was to propose a low cost, noninvasive screening system to detect CAD in an ICU patient. It is noticed that the study discloses remarkable achievement in sensitivity and specificity scores with the SVM classifier.

Paradkar et al. [45] employed an SVM-based classifier in order to detect CAD patients using PPG signals. Their proposed methodology extracts relevant features from the morphological structure of PPG signals. The supervised learning method SVM obtained a high performance evaluation in terms of sensitivity and specificity.

Banerjee et al. [46] proposed a methodology to detect CAD from PCG and PPG signals. The authors extract spectral features from the signals, and in the end, they employed an SVM classifier in order to detect CAD patients. Their study reveals that their proposed methodology achieved 80% classification accuracy using the SVM classifier.

According to [27], SVM is the most commonly used classifier in different types of applications in medical research. It can classify the input samples, whereas the input samples are linearly separable. However, the study implements SVM to classify the parameters of CAD detection and they discover that the training error is minimized from 0.31% to 0.05% while training the dataset using SVM. This classifier is not enough to handle a large dataset.

#### 3.4.4. Fuzzy Rule-Based Learning Technique (FR)

This learning technique is based on fuzzy rule-based system that could noninvasively predict CAD based on clinical parameters of the CAD risk index. Marateb and Goudarzi [19] proposed a fastest fuzzy rule-based classification technique in the context of CAD detection where they set the rules using clinical attributes and process it for CAD and normal differentiation. The study successfully obtained 84% accuracy in the classification procedure.

Mohammadpour et al. [32] used fuzzy rule-based classification to predict CAD. According to the authors, fuzzy classification based on the if-then rule successfully achieved accuracy of 92.8%, whereas 91.9% obtained classification accuracy using equation. However, fuzzy rules were evaluated to reduce space dimension for training the classifier in order to improve the accuracy.

Pal et al. [47] formulated fuzzy rules based on medical experts' approach for CAD detection. The proposed methodology is built to assist doctors to identify the risk and prediction of CAD patients. Ten rules are created and each block of the rule is representing a module and each module is containing one risk factor. However, the developed system leads to 95.85% sensitivity and 83.33% specificity in CAD risk computation.

#### 3.4.5. Other Techniques

In literature, except the discussed methods in medical research, various other methods for CAD classification have been developed using other learning and data mining techniques, for instance, random forest [48, 49], decision tree [50, 51], clustering [52, 53], and Gaussian mixture model [54].

Random forest (RF) classifier is a ombination of multiple tree predictors which frequently used for big data analysis. RF is an ensemble learning technique for classification, regression, and for other tasks.

Banerjee et al. [55] conducted experiment using the random forest classifier. Their study extracts time-frequency features from PCG signals in order to classify heart diseases, and it successfully achieved high performance in its applied methods.

SVM, NN, KNN, and RF classifiers were used for heart failure classification by [49]. They performed comparison analysis between classifiers and disclosed that the RF classifier stands out with 100% accuracy. Furthermore, the RF classifier successfully achieved significant advantages among other implemented classifiers in the study. The RF classifier was applied by [56, 57] to differentiate normal and abnormal heartbeats and successfully achieved 92.2% and 93% success, respectively.

The decision tree (C4.5) classifiers are non-parameteric supervised learning technique used for classification and regression.The aim of this technique is to create a model that predicts the value of a target variable by learning simple decision rules. Baihaqi et al. [58] performed an experimental research to diagnose CAD using C4.5, and they successfully obtained accuracy of 78.95%. However, studies reveal that the C4.5 classifier is not a promising approach for continuous features [59, 60]. Thus, a technique that uses the C4.5 classifier considers only small dataset. For instance, in [49], the large dataset was considered for the detection of heart disease; in that case, the RF classifier performed better than C4.5. Meanwhile, the combination technique was used in [61], and the authors noticed that the bagged decision tree classifier obtained remarkable progress to discriminate the classes of the feature set.

According to [54], the methodology was developed using the Gaussian mixture model (GMM) unsupervised learning of classification where it returns remarkable performance with 99.42% accuracy over ten folds. The study also revealed that using the GMM low probability error of 3.0700 × 10^−5^ was obtained as an upper bound on the classification error. The GMM is used in [10], to reestimate attributes from the dataset in order to calculate the class mean and covariance matrix for a priori probability estimation. The GMM model is used for testing the data and noting the performance of unsupervised learning, while classifying CAD resulted in the highest accuracy of 96.8%.

Clustering methods are unsupervised learning that are widely used with different supervised learning algorithms in recent studies [53, 62]. According to [63], they combined the NN classifier with unsupervised learning methods taking into account that the accuracy obtained is much better than single NN classifier. Other studies [64, 65] in the same direction used clustering technique with a linear discriminate classifier for heart patients, and it reveals remarkable performance results which are reliable and efficient for real-world application.

## 4. Discussion

Data mining techniques play a major role in medical systems, which will provide the major contribution to enhance the medical field. This study presents coronary artery disease classification review on different methods of data mining and artificial intelligence. Furthermore, we observed that from literature, there are two types of parameters used in CAD classification. However, we represent features in this study as parameter set A and parameter set B which are signal features (Table 1) and patient clinical data (Table 2), respectively. However, Table 3 describes review of state-of-the-art classifiers and their effectiveness. Furthermore, we proposed two workflows (Figures 1 and 2) for the evaluation process of future works for beginners in this field. Figure 1 shows that preprocessing and feature extraction are the most important phases for parameter set A, and Figure 2 depicts that it is not necessary to go through preprocessing and feature extraction stages for parameter set B. However, remaining flows are the same for both diagrams.

In literature, we found that there is still a room for improvement in CAD classification. ECG is a noninvasive technique used to diagnose CAD patients, and ECG signal does not provide the proper information that is required [5, 6, 8] though it is necessary to obtain accurate feature from the ECG. This limitation may also lead to a serious heart disease. Therefore, a suitable method for hidden factor extraction from ECG signal is very intricate due to the irregular shape of biosignals. Some studies like [66, 68] reported that feature extraction method is unable to calculate accurate values of unmasked attributes of the ECG signal. Furthermore, the usage of the small dataset for classification may diagnose misclassification and it is also necessary to avoid small dataset for classification in order to overcome the error rate [9, 37, 69].

## 5. Conclusion

In this paper, we reviewed automated CAD classification state-of-the-art methods. In literature, we found that SVM classifier performance is better than another classifier for automated detection of CAD. Our study proposed two workflows for parameter sets A and B in which we analyzed that two stages are most important while using parameter set A. Furthermore, we also suggest that performance of the classifier also relies on dataset's nature and size.

## Figures and Tables

**Table 1:** Parameters of ECG.

Features	Description
SDNN	Standard deviation of normal RR intervals
SDSD	The standard deviation of successive RR interval difference
RMSSD	Square root of the mean of the sum of the squares differences between adjacent normal intervals
QRS duration	Area under peak
Mean	Average values

**Table 2:** Patient clinical data [66].

Features	Description	Ranges
Age	Age (in years)	30–86
Gender	1: male; 0: female	0–1
HTN	Hypertension, 0: no; 1: yes	0–1
RBS	Random blood sugar	57–180
Chest pain type	0: nonspecific chest pain	0–2
	1: atypical chest pain	
	2: typical angina	
HT	Height (cm)	133–188
WT	Weight (kg)	33–110
DBP	Diastolic blood pressure (mmHg)	46–110
SBP	Systolic blood pressure (mmHg)	100–170
CAD	Coronary artery disease	0: no; 1: yes

**Table 3:** Review of state-of-the-art classifiers and their effectiveness.

Work	Feature set	Classifiers	Effectiveness
[8]	A	Optimized SVM	Accuracy = 99.2% Sensitivity = 98.43% Specificity = 100%
[66]	B	NN	Accuracy = 88.4%
[10]	A	KNN	Accuracy = 96.8% Sensitivity = 100% Specificity = 93.7%
[9]	A	LS-SVM	Accuracy = 99.7% Sensitivity = 99.6% Specificity = 99.8%
[27]	A	SVM	Accuracy = 79.71%
[7]	A	LS-SVM	Accuracy = 100%
[19]	B	Fuzzy rule	Accuracy = 84% Sensitivity = 79% Specificity = 89%
[32]	B	Fuzzy rule	Accuracy = 92.8%
[58]	B	Fuzzy rule	Accuracy = 81.2%
[67]	B	Fuzzy rule and ensemble classifier	Accuracy = 84.44%
[55]	A	Random forest	Sensitivity = 80% Specificity = 90%
[44]	A	SVM with RBF	Sensitivity = 73% Specificity = 87%
[45]	A	SVM	Sensitivity = 85% Specificity = 78%
